# Prospective evaluation of indirect costs due to acute rotavirus gastroenteritis in Spain: the ROTACOST study

**DOI:** 10.1186/1471-2431-11-81

**Published:** 2011-09-14

**Authors:** Marta Bouzón-Alejandro, Lorenzo Redondo-Collazo, Juan Manuel Sánchez-Lastres, Nazareth Martinón-Torres, José María Martinón-Sánchez, Federico Martinón-Torres

**Affiliations:** 1 Área de Pediatría, Hospital Clínico Universitario de Santiago de Compostela, Spain; 2 Grupo Gallego de Genética, Vacunas e Investigación Pediátrica (G3VIP), Instituto de Investigación Sanitaria de Santiago, Spain; 3 Red Gallega de Investigación Pediátrica, Spain; 4 Centro de Salud de Chapela, Pontevedra, Spain; 5 The ROTACOST research team is composed of members of the Galician Pediatric Research Network (ReGALIP

## Abstract

**Background:**

The effect of rotavirus in developed countries is mainly economic. This study aimed to assess the indirect costs induced by rotavirus acute gastroenteritis (RVAGE) in Spain.

**Methods:**

A prospective observational study was conducted from October 2008 to June 2009. It included 682 children up to 5 years of age with acute gastroenteritis (AGE) who attended primary care (n = 18) and emergency room/hospital settings (n = 10), covering the regions of Galicia and Asturias (North-west Spain). All non-medical expenses incurred throughout the episode were recorded in detail using personal interviews and telephone contact.

**Results:**

Among the 682 enrolled children, 207 (30.4%) were rotavirus positive and 170 (25%) had received at least one dose of rotavirus vaccine. The mean (standard deviation) indirect cost caused by an episode of AGE was estimated at 135.17 (182.70) Euros. Costs were 1.74-fold higher when AGE was caused by rotavirus compared with other etiologies: 192.7 (219.8) Euros vs. 111.6 (163.5) Euros (p < .001). The costs for absenteeism were the most substantial with a mean of 91.41 (134.76) Euros per family, resulting in a loss of 2.45 (3.17) days of work. In RVAGE patients, the absenteeism cost was 120.4 (154) Euros compared with 75.8 (123) for the other etiologies (p = .002), because of loss of 3.5 (3.6) vs 1.9 (2.9) days of work (p < .001). Meals costs were 2-fold-higher (48.5 (55) vs 24.3 (46) Euros, p < .001) and travel costs were 2.6-fold-higher (32 (92) vs 12.5 (21.1) Euros, p = .005) in RVAGE patients compared with those with other etiologies. There were no differences between RVAGE and other etiologies groups regarding costs of hiring of caregivers or purchase of material. Patients with RVAGE were admitted to hospital more frequently than those with other etiologies (47.8% vs 14%, p < .001).

**Conclusions:**

Rotavirus generates a significant indirect economic burden. Our data should be considered in the decision-making process of the eventual inclusion of rotavirus vaccine in the national immunization schedule of well developed countries.

## Background

Rotavirus is the leading cause of acute gastroenteritis (AGE) in infants and children under 5 years of age, causing 600,000 deaths worldwide [1]. In developed countries, death by rotavirus is very rare, but infection by rotavirus is an important cause of morbidity, and it represents a high cost. At the age of 5 years, almost all children will have suffered an episode of acute rotavirus gastroenteritis (RVAGE), 1 of each 5 will need a visit to the physician, 1 of 65 will be hospitalized and approximately 1 of 293 will die [1-5]. In the last 20 years, the mortality rates by rotavirus have decreased in the more developed countries, but improvement in hygienic-healthy conditions has not modified the global incidence of the disease, and therefore, vaccination is the most efficacious measure to prevent this illness [6-9].

In the USA, it has been estimated that rotavirus infections generate direct sanitary costs of 250 million of dollars per year and the indirect costs are more than a billion dollars. Despite the fact that rotavirus gastroenteritis is currently considered to be the most common pediatric disease in the European Community preventable by vaccination, specific data about the indirect economic burden generated by rotavirus in Europe is limited [7,10-15]. In Spain, economic data on rotavirus are even more scarce, and its impact in terms of indirect costs is almost unknown [6,16-20]. The REVEAL study considered both direct and indirect costs, and compiled data from 7 countries including Spain [20]. Another single centre hospital-based study performed by our group showed that the indirect costs generated by RVAGE were 2.6-fold higher than those induced by other etiologies [18,19].

The aim of the ROTACOST study was to prospectively determine the indirect economic burden of AGE caused by rotavirus in a broad population including not only hospital-care but also emergency and primary care settings.

## Methods

A prospective, observational multicentric study was conducted from October 2008 to June 2009 using a pediatric research network (ROTACOST study group -ReGALIP - http://www.regalip.org) that included primary, emergency and hospital care settings. Any patient up to 5 years old seeking care because of an AGE episode was considered eligible for the study if they fulfilled the following conditions: a) 3 or more bowel motions less consistent than usual, associated or not associated with vomiting, in a 24-hour period; b) symptoms must have occurred within 7 days of enrollment, preceded by a 14-day symptom-free period; and c) an episode of AGE must not have occurred in the 2 weeks prior to the onset of current symptoms. All nonmedical expenses from the start to the end of the diseases were recorded in detail using personal interviews and telephone contact. A fecal immunoassay for rotavirus detection in a fecal sample was performed in all included patients (VIKIA Rota-Adeno^®^, Biomerieux). This study was approved by the reference ethics committee, Comité Etico de Investigación de Galicia (ref 2009/039). All parents or guardians received information about the study and signed an informed consent before study entry.

### Data collection

Clinical, epidemiological and economic data were recorded and uploaded through a specific web site. The data collection was performed in 2 phases: first, personally during a contact visit; and second, by either phone or a planned visit once the acute episode had finished. During the first contact, the parents received specific information and guidance for data collection until the second survey/visit.

### Methods to estimate indirect expenses

All expenses incurred before, during, and after patient contact and study entry, from the start of symptoms to total recovery of the child, were recorded. Expenses during care or hospitalization for patient diets, materials, or medication were considered as part of direct (hospital) costs, and were not counted as indirect expenses. Priority was always given to the exact cost, when known by the parents. Costs in the following areas were evaluated and calculated as specified:

1. Work

Estimated cost of work lost was calculated by the following order: (a) using the exact amount, if the person interviewed knew the exact rate per hour, (b) based on the annual/monthly/weekly earnings, the mean amount earned per hour, considering the numbers of hours worked per week, and the number of hours lost, and (c) based on the Spanish minimum mandatory salary for the year 2008 (Royal Decree 1763/2007), i.e., 28.42 Euros per day. Both spouses were considered if they both worked.

2. Travel

Any journey to the physician's office, hospital, pharmacy, or related to or caused by the child's condition was considered. Distance traveled was registered, and the cost of traveling by car was calculated by applying article 8.A.2 of the Regulations of the Personal Income Tax, approved by the Royal Decree 1775/2004, which sets a cost of 0.19 Euros per kilometer traveled.

3. Caregiver

This included day nursery or caregiver expenses, but only when contracted for care of the other siblings.

4. Meals

Expenses incurred by meals outside the home because of the disease of the child. The number of meals and the cost per meal were recorded.

5. Material

This included any expenses derived from the purchase of oral rehydration or similar solutions, antidiarrheal agents or other drugs prescribed by the physician, additional number of diapers used and their cost, specific special diets (e.g., lactose-free), topical creams, or any other materials purchased during the time of the disease that were adequately explained by the parent or guardian.

### Statistical analysis

When appropriate, data are presented as mean (standard deviation). Patients were divided into "rotavirus acute gastroenteritis" (RVAGE) and "other etiologies" groups for comparison purposes. Normal distribution of the data was assessed with the Kolmogorov-Smirnov test, and the Mann-Whitney or Student's t test was used accordingly. Multiple linear regression analysis was performed to adjust for the effect of the etiology (rotavirus vs another etiology), admission, age and sex of patients in the generated costs. A value of p < 0.05 was considered significant. Statistical analyses were performed using SPSS version 17.0 (SPSS Inc., Chicago Illinois, USA).

## Results

A total of 765 children were invited to participate in the study. Fifteen children declined to participate, 56 did not complete the study and 12 were withdrawn because of missing data in any of the study end-points. Finally, 682 patients suffering from AGE with a mean age of 18.5 (12.9) months were included (Table 1). A total of 207 (30.4%) children were positive for rotavirus and 170 (25%) had received at least 1 dose of rotavirus vaccine. Each child required a mean of 2.1 (1.2) medical visits during the disease, and 163 (23.9%) patients required hospital admission, with a mean stay of 4.4 (2.1) days. Patients with RVAGE were admitted to hospital more frequently than patients with other etiologies (47.8% vs 14.0%, p < 0.001) (Table 1).

**Table 1:** Summary of patient characteristics

	GLOBAL (All etiologies) (n = 682)	Gastroenteritis by rotavirus (n = 207)	Gastroenteritis by other etiologies (n = 428)	P
**Age (months)**				0.563
0-6 months	111 (16.3%)	28 (13.5%)	75 (17.5%)	
7-12 months	141(20.7%)	42 (20.3%)	89 (20.8%)	
13-24 months	253 (37.1%)	81 (39.1%)	152 (35.5%)	
25-36 months	109 (16.0%)	38 (18.4%)	67 (15.7%)	
37-60 months	68 (10.0%)	18 (8.7%)	45 (10.5%)	
**Sex (male %)**	383 (56.2%)	116 (56.0%)	241 (56.3%)	0.949
**Rotavirus vaccination **(complete or partial vaccination)	178 (26.1%)	11 (5.3%)	159 (37.1%)	< 0.001
**Maximum number of depositions in 24 hours**	6.9 ± 3.4 (6.0)	8.0 ± 4.0 (7.0)	6.3 ± 3.0 (6.0)	< 0.001
**Number of times needed medical care**	2.1 ± 1.2 (2.0)	2.3 ± 1.3 (2.0)	2.0 ± 1.2 (2.0)	< 0.001
**Gastroenteritis episode duration (days)**	7.3 ± 3.5 (7.0)	7.6 ± 2.9 (7.0)	7.2 ± 3.7 (6.0)	0.132
**Hospital admission**	163 (23.9%)	99 (47.8%)	60 (14.0%)	< 0.001
**Length of hospital stay (days)**	4.4 ± 2.1 (4.0)	4.5 ± 2.1 (4.0)	4.4 ± 2.1 (4.0)	0.850

"Global" represents all patients included in the study. P values are provided for comparison between rotavirus and other etiologies subgroups.

The mean indirect costs caused by an episode of AGE was estimated at 135.17 (182.7) Euros. Costs were 1.74-fold higher when AGE was caused by rotavirus compared with other etiologies (192.7 [219.8] Euros vs 111.6 [163.5] Euros, p = 0.001) (Table 2). Costs derived from absenteeism were the more substantial (64.5% of total costs), with a mean of 91.41 (134.76) Euros per family, resulting from the loss of 2.45 (3.11) days of work. In RVAGE patients, the absenteeism cost was 120.4 (154) Euros compared with 75.8 (123) Euros for those with other etiologies (p = .002), because of the loss of 3.5 (3.6) vs 1.9 (2.9) days of work (p < .001). Costs from purchase of materials were estimated at 34.4 (51.35) Euros per family, and represented 25.4% of overall costs. The remaining 36.9% of indirect costs were generated by the following: 5.5 (7.5) meals outside the home with a cost of 33.3 (51.4) Euros; hiring of specific caregivers with a cost of 14.7 (41.40) Euros; and traveling expenditure of 18.6 (55) Euros for a mean of 56.1 km (113.5) in each case. Meals costs were 2-fold-higher in RVAGE compared with those in other etiologies (48.5 [55] Euros vs 24.3 [46] Euros, p < .001). Travel costs were 2.6-fold-higher in RVAGE compared with those in other etiologies (32 [92] Euros vs 12.5 [21.1] Euros, p = .005). There were no differences between groups regarding costs of hiring of caregivers or purchase of material (Table 2).

**Table 2:** Indirect costs

	GLOBAL *Mean ± SD (Median)*	ROTAVRIUS *Mean ± SD (Median)*	OTHERS *Mean ± SD (Median)*	p
**Job**				
N° of hours of lost work	19.60 ± 25.36 (10)	28.1 ± 28.9 (20)	15.6 ± 22.9 (8)	< 0.001
N° of days of lost work	2.45 ± 3.17 (1,25)	3.5 ± 3.6 (2.5)	1.9 ± 2.9 (1)	< 0.001
Costs (€)	91.41 ± 134.76 (40)	120.4 ± 154.1 (72.5)	75.8 ± 123.3 (28.4)	0.002
**Travels**				
N° of Kms.	56.08 ± 113.46 (20)	79.3 ± 158.7 (26.5)	46.7 ± 92.8 (18)	0.050
Costs of travelling (€)	18.66 ± 55.30 (7)	32.1 ± 92.1 (14.8)	12.5 ± 21.1 (5)	0.005
**Caregiver (€)**	14.70 ± 41.40 (0)	14.8 ± 44.4 (0)	15.7 ± 41.4 (0)	0.873
**Meals**				
N° of meals outside home	5.47 ± 7.46 (2)	8.2 ± 7.8 (6)	4.1 ± 7.1 (1)	0.003
Cost meals (€)	33.33 ± 51.35 (10)	48.5 ± 55.9 (40)	24.3 ± 46.8 (0)	< 0.001
**Material (€)**	34.4 ± 30.7 (27,7)	34.3 ± 25.9 (28.4)	34.0 ± 33.5 (27.0)	0.921
Oral Serum (€)	9.88 ± 9.95 (8,90)	10.2 ± 12.3 (8.5)	9.7 ± 9.03 (8.6)	0.617
Special milk (€)	11.52 ± 21.01 (3,15)	10.2 ± 18.9 (0)	12.6 ± 23.3 (3.3)	0.305
Anti-diarrheal (€)	3.46 ± 5.21 (0)	3.5 ± 5.8 (0)	2.7 ± 4.6 (0)	0.224
Extra diapers (€)	11.89 ± 12.70 (10)	12.3 ± 9.7 (10)	11.2 ± 14.1 (8.5)	0.368
Creams (€)	4.43 ± 6.38 (3)	3.2 ± 4.4 (0)	4.9 ± 7.1 (3)	0.016
Other materials (€)	7.0 ± 12.74 (2,53)	6.01 ± 10.1 (0)	7.8 ± 4.1 (3)	0.197
**Other expenses (€)**	14.34 ± 92.43 (0)	12.1 ± 39.3 (0)	17.3 ± 19.5 (0.8)	0.711
**TOTAL INDIRECT COSTS (€)**	**135.17 ± 182.70 (67)**	**192.7 ± 219.8 (99,5)**	**111.6 ± 163.5 (54,3)**	< 0.001

*"Global" represents all patients included in the study. P values are provided for comparison between rotavirus and other etiologies subgroups*.

Indirect costs were also significantly higher in hospitalized patients compared with those in non-hospitalized patients (293.4 [261.2] Euros vs 85.5 [110.4] Euros, p < .001).

## Discussion

The ROTACOST study showed that there is an outstanding burden generated by AGE in the North-west of Spain in terms of indirect costs, mainly because of the absenteeism of parents. When rotavirus is the etiological agent, costs are 75% higher compared with those for other etiologies, and these costs account for one third of the Spanish minimum official salary.

Few previous studies in this setting were prospective, focused on indirect costs and included all types of patients from primary care to hospital care [18-21]. Only Lee et al. have compiled such a comprehensive collection of indirect costs in the USA setting [21]. The REVEAL study assessed both the direct and the indirect costs of RVAGE in 3 different health care settings (hospital, emergency department, and primary care) [20]. Absenteeism costs were also the highest in every country surveyed in the REVEAL study, as a consequence of a variable work loss that ranged from 2.3 days in France to 7.5 days in the United Kingdom. Other studies with similar objectives excluded 25% to 80% of the expenses sources taken into account in these 3 studies [22-25].

Prior to rotavirus vaccine availability in Spain, we performed a study with the same methodology as the current study but restricted it to one single centre [18]. We found that rotavirus cases accounted for 60% of the total cases and an event of RVAGE was estimated to cost €427, twice the cost of an episode due to any other etiology. The differences between our previous study and the present study may be mainly related to the implementation of rotavirus vaccine and an actual decline in the number and severity of RVAGE cases. In addition, less severe patients have been now included through the primary care recruiting network.

According to recent epidemiological data regarding rotavirus disease in Spain, approximately 310,000 cases of rotavirus-induced diarrhea occur every year in children under 5 years of age, causing 14,000 hospitalizations [16,17,26,27]. Consistent with our study, only the indirect costs of rotavirus in patients requiring hospital admission would amount to €3 million every year. A recently published model of a universal rotavirus vaccination program applied to Spain using cost data consistent with our study, showed that more than 136,000 cases of RVAGE and €60 million (€38 from a societal perspective) in expenses could be avoided per birth cohort [27].

The main limitations in our study are those common to multicentric and economical studies based on the survey approach. Differences in methodology prevented direct comparison with some other studies mainly because of not including/specifying all the sources detailed in our study. Previous experience of the main team with the same protocol [18], appropriate training of the participating subinvestigators and a very conservative approach applied are indicative that the obtained results are reliable and demonstrative -if not under-estimative- of the actual burden of rotavirus in terms of indirect costs.

## Conclusions

In conclusion, rotavirus disease represents a significant economic burden in terms of indirect costs in our country. This fact reinforces the interest of rotavirus vaccination in developed countries and it should be taken into account in the assessment and decision-making process of the inclusion of rotavirus vaccine into national immunization programs

## Competing interests

Dr. F. Martinon-Torres has received research grants and/or honoraria as consultant/advisor and/or speaker from GlaxoSmithKline, Sanofi Pasteur MSD, Pfizer Inc, Wyeth, Novartis, and Medimmune Inc.

## Authors' contributions

FMT, MBA, LRC, JSL and JMS conceived the study and participated in its design and coordination, and helped to draft the manuscript. FMT created and directed the network. MBA performed the main field work. (5) The ROTACOST research team performed the recruitment of patients and data collection. All signing authors read and approved the final manuscript.

## Pre-publication history

The pre-publication history for this paper can be accessed here:

http://www.biomedcentral.com/1471-2431/11/81/prepub
